# Oral mucosal lesions among dental outpatients in Al-ahsa, Saudi Arabia: prevalence, clinical patterns and associated determinants: a cross-sectional study

**DOI:** 10.3389/froh.2026.1844835

**Published:** 2026-07-20

**Authors:** Mousa Haney AlSleem, Hussain Adel Alghafli, Abdullah Othman Alasafirah, Hussain Mohammed Alkhames, Ahmed Khalid Elhag, Hatim Mohammed Almahdi

**Affiliations:** 1 Oral and Maxillofacial Surgery and Diagnostic Sciences Department, College of Dentistry, King Faisal University, Al-Ahsa, Saudi Arabia; 2 Department of Restorative Dental Sciences, College of Dentistry, King Faisal University, Al-Ahsa, Saudi Arabia

**Keywords:** cross section study, normal variant, oral mucosal lesions, prevalence, Saudi arabia

## Abstract

**Background/objectives:**

Oral mucosal lesions (OMLs) are common worldwide and can disrupt daily activities by affecting chewing, swallowing and speech. This study aimed to determine the prevalence of OMLs among Saudis attending a dental clinic in Al-Ahsa, Saudi Arabia, identify their clinical patterns and examine associated determinants.

**Methods:**

This cross-sectional study was conducted between May and September 2023. All Saudis the King Faisal University dental complex were invited to participate. OMLs were diagnosed based on clinical features, and well-trained, calibrated examiners carried out the assessments.

**Results:**

A total of 462 patients participated, including 282 (61%) males, with a mean age of 23.01 ± 15.39 years (SD). The majority were older than 18 years (272, 58.9%). OMLs were identified in 214 participants (46.3%). Just over half of those affected had a single lesion (54.7%), while 42.5% presented with two lesions. The most common lesions observed were linea alba (41, 19.2%), melanin pigmentation (40, 18.7%) and bone exostosis (22, 10.3%). OMLs were more prevalent among males (148, 69.2%) and participants older than 18 years (183, 85.5%; *p* ≤ 0.01). The binary logistic regression model indicated that age, educational level and occupation were significant predictors (*p* < 0.05). Participants aged ≤ 18 years had significantly lower odds of OMLs than those older than 18 years (OR = 0.06, CI: 0.034–0.111, *p* ≤ 0.001). For educational level, illiterate individuals had 6.1 times higher odds of having OMLs (OR = 6.11, CI: 1.17–32.02, *p* < 0.03). Regarding occupation, employed individuals had 2.2 times higher odds of OMLs than unemployed individuals (OR = 2.22, CI: 1.13–4.37, *p* ≤ 0.02).

**Conclusions:**

OMLs were observed in 46.3% of dental attendees, with the most frequently noted types being linea alba, oral melanin pigmentation and bone exostosis. These lesions were more common among males, adults, individuals with lower literacy and employed participants. These findings indicate the common occurrence of benign oral mucosal conditions in this population and suggest that clinicians should maintain regular vigilance during dental examinations.

## Introduction

The oral mucosal lining serves as a protective barrier, preventing mechanical trauma, the entry of microorganisms and the absorption of carcinogenic agents (1). A lesion is a broad term defined as ‘any pathological or traumatic discontinuity of tissues or loss of function of any part' (2). Oral Mucosal Lesions (OMLs) are any abnormal changes (red, pigmented or ulcerative) or any swelling of the oral mucosal surface (3).

OMLs are common across many populations worldwide and can interfere with daily personal and social activities. They may cause severe pain that limits food intake, impairs oral hygiene and makes swallowing and speaking difficult, ultimately affecting oral health-related quality of life (4).

Although some OMLs are benign and require no treatment, others may present with significant pathology and require immediate intervention, such as oral potentially malignant disorders, which may progress to malignancy, including oral cancer, often beginning with changes such as ulceration (5).

Globally, the prevalence of OMLs varies significantly across countries and even districts, ranging from 4.9% to 64.7% (1). Studies conducted in different regions of Saudi Arabia have also shown variation in the types of OMLs observed. Al-Mobeeriek et al. estimated the prevalence of OMLs in the central region of Saudi Arabia at 15%, with most cases presenting as Fordyce granules. However, Al Wayli et al. found that pyogenic granuloma was the most common lesion among Saudi females (6). A recent study conducted in Riyadh reported a prevalence rate of 28.3% for OMLs, with abscesses (45%) and linea alba (35%) being the most frequently observed lesions (7).

Detrimental habits, irregular or sharp teeth, ill-fitting prostheses and poor oral hygiene are among the factors that may contribute to the occurrence of OMLs. Many oral lesions, both pathological and congenital, are also associated with systemic conditions such as genetic predispositions, skin diseases and blood disorders (8).

Understanding the distribution of OMLs, their epidemiology and contributing factors is necessary to support primary prevention, early detection and appropriate treatment, as well as to prevent the malignant transformation of potentially malignant lesions. As far as we know, no study has been conducted to assess the prevalence of OMLs in the Al-Ahsa region of the Eastern Province, Saudi Arabia.

This study aimed to estimate the prevalence of OMLs among dental attendees visiting the Dental Complex at King Faisal University (KFU) in Al-Ahsa, Saudi Arabia. In addition, it aimed to identify the types and sites of OMLs and to explore their relationships with sociodemographic factors and general health.

## Materials & methods

### Study area, population and design

A cross-sectional study was conducted in the Al-Ahsa region of the Eastern Province, Saudi Arabia, to estimate the prevalence of OMLs among Saudi dental attendees and determine associated factors. The study was carried out at the Dental Complex of KFU between May 2023 and September 2023. The KFU Dental Complex is the largest dental facility in the Al-Ahsa region. It comprises 100 dental units and is supported by a multidisciplinary team of specialists, general practitioners, dental assistants and administrative staff across various clinical departments. The complex provides care to over 4,000 patients annually and serves as the primary site for clinical training of dental students at KFU (9).

Al-Ahsa Governorate represents 24% of the total area of the Kingdom of Saudi Arabia and approximately 68% of the Eastern Province, with Al-Hofuf as its capital. It has a population of about 1.104 million, with the majority residing in urban areas. The governorate is divided into four districts: Al-Hofuf, Al-Mubarraz, Eastern and Northern towns (10).

### Sampling method and sample size

The sample size was calculated using Cochran's formula: *n* = Z^2^·p(1−p)/d^2^. In this formula, Z denotes the standard normal value for a 95% confidence level (1.96), p represents the expected prevalence of oral lesions, and d is the acceptable error (0.05). Because no prior prevalence estimates were available for this population, a conservative *p*-value of 0.5 was chosen. The calculation determined that a minimum of 385 participants was necessary. A total of 462 Saudi attendees at the KFU Dental Complex were ultimately recruited, exceeding the calculated minimum by approximately 20%.

### Data collection

The data collection process was divided into two main phases: the first involved an interview, during which examiners collected participants' personal information. The second phase consisted of a clinical examination.

### Training and calibration of examiners

Training and calibration of the four examiners were conducted at two-week intervals. A set of 25 photographs of the most common OMLs was reviewed by the four examiners over successive days. The photographs represented the full range of OMLs expected to be assessed during the survey. Reviews were repeated until acceptable consistency was achieved, as confirmed using Cohen's kappa. Intra- and inter-examiner reliability were 100% and 81.8%, respectively, indicating substantial agreement. The examination followed the WHO protocol for OMLs assessment.

### Clinical examination

The clinical examination was conducted using dental examination instruments, including gloves, masks, cotton and tongue depressors. The datasheet recorded OMLs and their intraoral sites. The oral mucosa and surrounding soft tissues were examined in the following sequence: labial mucosa and labial sulci (upper and lower), labial commissures and buccal mucosa (right and left), tongue (dorsum, ventrum and margins), floor of the mouth, hard and soft palate and alveolar ridges/gingiva (upper and lower).

### Criteria for diagnosis of OMLs

The diagnosis of specific OMLs was based on clinical features listed by the World Health Organization (WHO), using checkboxes to record the presence or absence of each condition. An OML is defined as any abnormal change in the oral mucosal surface (red, pigmented or ulcerative) or any swelling (11). Developmental variations such as sublingual varix, geographic and fissured tongue and Fordyce granules were also included (3).

### Data management and analysis

Data were analysed using the Statistical Package for the Social Sciences (SPSS, IBM Corp., Armonk, NY, USA), version 30. Descriptive analyses were performed using frequencies and percentages for categorical variables and means and standard deviations for numerical variables. Cross-tabulations were conducted between the dependent and independent variables (demographic factors) to assess statistical significance using the chi-square (*χ*^2^) test. Effect size was evaluated using the phi coefficient and Cramer's V to determine the strength of association. Multivariate analysis was performed using binary logistic regression.

### Ethical consideration

The Research Ethics Committee at KFU granted ethical approval for the study (KFU-REC-2023-SEP-ETHICS1105). Written informed consent was obtained from all adult participants, while for children, parents were provided with written and verbal information about the study. Participant confidentiality and anonymity were strictly maintained in accordance with the principles of the Declaration of Helsinki.

## Results

A total of 462 patients participated, including 282 (61%) males, with a mean age of 23.01 ± 15.39 years (SD). Most participants were older than 18 years (272, 58.9%), while 190 (41.1%) were aged ≤18 years. The majority resided in urban areas (353, 76.4%) and were employed (398, 86.1%). Most participants were educated (450, 97.4%; Table 1).

**Table 1:** Frequencies and percentages of demographic characteristics of the participants.

Variables		Frequency (%)
Age group	≤18 years	190 (41.1)
	>18 years	272 (58.9)
Gender	Male	282 (61.0)
	Female	180 (39.0)
Health concern	No	403 (87.2)
	Yes	59 (12.8)
Residence	Rural	109 (23.6)
	Urban	353 (76.4)
Occupation	Employed	398 (86.1)
	Unemployed	64 (13.9)
Educational level	Illiterate	12 (2.6)
	School	293 (63.4)
	University	157 (34.0)
Presence of oral lesions	No	248 (53.7)
	Yes	214 (46.3)

OMLs were detected in 214 participants (46.3%). Of those affected, 54.7% had a single lesion, while 42.5% had two lesions. The most common lesions observed were linea alba (41, 19.2%), melanin pigmentation (40, 18.7%) and bone exostosis (22, 10.3%; Table 2).

**Table 2:** Frequency (N) and percentage (%) of OMLs types among participants.

Characteristics	OML	N (%)
Normal variants	Leukoedema	4 (1.9)
	Fordyce granules	10 (4.7)
	Linea alba	41 (19.2)
	Bone exostosis	22 (10.3)
Tongue lesions	Coated tongue	5 (2.3)
	Hairy tongue	14 (6.5)
	Geographic tongue	8 (3.7)
Lichenoid reactions	Lichen planus	3 (1.4)
Inflammatory lesions	Herpes labialis	3 (1.4)
	Candidiasis	5 (2.3)
Reactive lesions	Frictional keratosis	13 (6.1)
	Traumatic fibroma	3 (1.4)
Pigmented lesions	Melanin pigmentation	40 (18.7)
Ulcers	Aphthous ulcers	12 (5.6)
White lesions	Leukoplakia	2 (0.9)
Others		14 (6.5)

The most common site in the oral cavity was the oral mucosa (98, 45.8%), followed by the alveolar ridge/gingiva (57, 26.6%), dorsal surface of the tongue (23, 10.7%), ventral surface of the tongue (6.5%), floor of the mouth (6.1%), hard/soft palate (2.3%) and lateral border of the tongue (1.9%; Table 3).

**Table 3:** Distribution of frequency (N) and percentage (%) of location and number of OMLs.

Location of oral lesion	N (%)
Oral mucosa	98 (45.8)
Alveolar ridge/gingiva	57 (26.6)
Tongue	41 (18.1)
Floor of the mouth	13 (6.1)
Hard/soft palate	5 (2.3)
Total	214 (100)
Number of oral lesions per patient	
One lesion	117 (54.7)
Two lesions	91 (42.5)
Three lesions	6 (2.8)
Total	214 (100)

Males presented more oral lesions (148, 69.2%) than females. Participants aged >18 years showed a higher prevalence of oral lesions (85.5%). Those residing in urban areas had a higher prevalence (75.2%) than those in rural areas. Most patients with oral lesions were employed (87.9%), and approximately half of the sample (50.4%) had a school-level education. Systemic diseases were reported in 14.5% of participants. Age-group analysis showed a higher frequency among participants aged >18 years (183, 85.5%) compared with those aged ≤18 years (31, 14.5%), with a statistically significant difference (*p* ≤ 0.01; Table 4).

**Table 4:** Distribution of frequencies (N) and percentages (%) of OMLs by gender, age and systemic diseases.

Variables		Presence of lesion		Total
		No	Yes	
Age group	≤18 years	159 (64.1)	31 (14.5)	190 (41.1)
	>18 years	89 (35.9)	183 (85.5) **	272 (58.9)
Gender	Male	134 (54.0)	148 (69.2) **	282 (61.0)
	Female	114 (46.0)	66 (30.8)	180 (39.0)
Health concerns	No	227 (91.5)	176 (82.2)	403 (87.2)
	Yes	21 (8.5)	38 (17.8) **	59 (12.8)
Residence	Rural	56 (22.6)	53 (24.8)	109 (23.6)
	Urban	192 (77.4)	161 (75.2)	353 (76.4)
Educational level	School education	181 (73.0)	112 (52.3)**	293 (63.4)
	University	59 (23.8)	98 (45.8)	157 (34.0)
	Illiterate	8 (3.2)	4 (1.9)	12 (2.6)
Occupation	Employed	210 (84.7)	188 (87.9)	398 (86.1)
	Unemployed	38 (15.3)	26 (12.1)	64 (13.9)

*p ≤ 0.05, **p ≤ 0.001.

 Table 5 shows the binary logistic regression identifying factors associated with the presence of oral lesions. After adjusting for gender, health concerns, residence, educational level and occupation, the model revealed that age, educational level and occupation were significant predictors (*p* < 0.05), while gender, health concerns and residence were not. Participants aged ≤18 years had significantly lower odds of oral lesions compared with those older than 18 years (OR = 0.06, CI: 0.034–0.111, *p* < 0.001).

**Table 5:** Multivariable binary logistic regression analysis of factors associated with OMLs among dental attendees.

Variables	B	AOR	95% CI	*p*
Age groups	−2.797	0.06	0.034–0.111	0.000
Gender	0.340	1.41	0.887–2.225	0.15
Health concerns	−0.236	0.79	0.415–1.505	0.47
Residence	−0.344	0.71	0.425–1.181	0.19
Educational level				
Educational level (illiterate)	1.811	6.11	1.168–32.01	0.03
Educational level (school)	0.590	1.81	1.045–3.116	0.03
Occupation	0.797	2.22	1.126–4.370	0.02

*p ≤ 0.05, **p ≤ 0.001.

Educational level was significantly associated with the presence of oral lesions (*p* = 0.03). Compared with university graduates, school-educated individuals had 1.8 times higher odds of oral lesions (OR = 1.81, CI: 1.05–3.12, *p* = 0.03). Illiterate individuals had 6.1 times higher odds (OR = 6.11, CI: 1.17–32.02, *p* = 0.03); however, the wide confidence interval indicates an imprecise estimate, likely reflecting the small number of illiterate participants in the sample. Regarding occupation, employed individuals had 2.2 times higher odds of oral lesions compared with unemployed individuals (OR = 2.218, CI: 1.126–4.370, *p* = 0.02). Gender (*p* = 0.15), health concerns (*p* = 0.47) and residence (*p* = 0.19) did not show statistically significant associations.

## Discussion

The present study found a high overall prevalence of OMLs among Saudi dental patients examined at the Dental Complex of KFU in Al-Ahsa. The prevalence observed is among the higher ranges reported in the literature and exceeds that reported in previous studies from other regions of Saudi Arabia. For example, a recent study conducted in Riyadh reported an OML prevalence of 28.3% (7).

The global prevalence of OMLs varies considerably across studies (5%–65%), reflecting differences in study populations, habits and methodologies used (1). These findings are similar to those reported in studies from Slovenia (61.6%), Chile (53%) and Yemen (56.8% (12–15);.

The lack of standardisation in research methods makes comparisons across studies challenging. As a result, the wide variation in reported prevalence is more likely attributable to methodological differences than to geographical location or the sociodemographic characteristics of the populations studied. Other contributing factors may include climate, health behaviours, ethnicity and genetic predisposition, all of which may influence disease susceptibility and presentation (16).

In this sample, the most frequently encountered lesions were linea alba, melanin pigmentation and bone exostosis. In contrast, a study conducted in Riyadh reported abscesses and linea alba as the most common lesions, with only low rates of pigmentation or exostosis (17). Another Saudi study conducted in a Riyadh clinic, which included only female participants, also reported different findings, with pyogenic granuloma and infections being the most common OMLs, followed by lichen planus (18). These differences suggest that OMLs patterns vary markedly across regions and populations in Saudi Arabia.

Similarly, a study from Kuwait reported that the second most common lesion after linea alba was generalised melanin pigmentation. Several other studies have also identified linea alba as one of the most frequently occurring lesions, consistent with our findings (19). In China, aphthous ulcers and burning mouth syndrome are the most common lesions. This variation may be due to multifactorial causes, including genetic background, stress and nutritional deficiencies (20).

The European studies showed lower OML prevalence rates of 10.3% and 7.5% among Portuguese and Norwegian populations, respectively, with the most common lesions often being reactive or normal variants [e.g., fibroepithelial hyperplasia in Portugal and exophytic and white lesions in Norway (21, 22);]. In North America, older data suggest a prevalence of approximately 27.9%, with higher rates observed among older adults, while systemic associations also varied across populations (23).

In this cohort, males presented with more lesions than females, consistent with a study from Nepal (24). In contrast, Al-Mobeeriek's survey in Riyadh reported a higher prevalence among females (25). These inconsistent patterns suggest that local factors, such as clinic referral patterns and gender-related health behaviours, may influence OML detection. In the study conducted in Portugal, there was a slightly higher proportion of females than males (22). Variability in gender distribution across studies may be attributed to behavioural factors such as higher rates of smoking and alcohol consumption among males, as well as generally poorer overall health status in males, which may also extend to oral health (26).

The prevalence of OMLs increases with age, a risk factor reported in several studies (19, 21, 22). Other factors also influence the oral environment and contribute to irritation and damage of the oral mucosal epithelium. Age-related changes in the oral cavity, such as atrophy of the oral mucosal epithelium, reduced mitotic activity and tissue regeneration, decreased cellular density and reduced levels of collagen and elastin, should be considered. These changes may also alter susceptibility of the oral flora to viral, fungal or bacterial infections, thereby contributing to the development of various lesions (27).

For OMLs, we found that urban residents and those with intermediate levels of education had higher odds of OMLs than rural residents and individuals with higher education levels. The Norwegian study similarly reported a higher prevalence of OMLs among individuals with low to intermediate education compared with those with higher education (21). Although individuals with higher education and income may often hold high-pressure jobs in competitive environments, which could contribute to stress-related conditions, this association should be interpreted with caution. Depressive symptoms in higher-income groups have also been linked to an increased risk of developing OMLs (28). However, the underlying mechanisms remain unclear and require further investigation.

The most common locations of OMLs in this study were the oral mucosa, followed by the alveolar ridge/gingiva and the tongue, findings similar to those of a study from Nepal. In Kuwait, approximately half of the lesions were found on the buccal mucosa, a pattern also reported in Portuguese studies (19, 22, 24). Most of these lesions are associated with mechanical friction or trauma, which commonly affects these areas, as well as the lateral border of the tongue.

Among the study participants, only 13.9% had systemic diseases. By contrast, the occurrence and distribution of systemic diseases in a Portuguese study was reported in 34.5% of the participants (22). These results are consistent with those in other studies (29, 30).

## Conclusion

OMLs were prevalent (46.3%) among dental attendees, with the most common types being linea alba, oral melanin pigmentation and bone exostosis. These lesions occurred more frequently in males, adults, illiterate individuals and employed participants. These findings highlight the high prevalence of benign oral mucosal conditions in this population and underscore the importance of clinicians maintaining regular vigilance during dental examinations.

### Strengths and limitations

This is the first study to comprehensively assess OMLs among dental attendees in the Eastern Province, addressing an important gap in the epidemiological literature on OMLs. The sample size (*n* = 462) is robust, and calibrated examiners used WHO criteria to ensure consistent diagnosis. A broad range of lesions was documented, and demographic and health factors were considered, allowing for a multifaceted analysis in line with established reporting recommendations for OML prevalence studies (16).

This study has several limitations that should be considered when interpreting the findings. The cross-sectional design limits causal inference, and the identified determinants should be interpreted as associated factors rather than causes. The exclusive sampling of dental clinic attendees, along with the sampling technique used, may limit the generalisability of the findings to the broader population. Although standardised diagnostic procedures were applied, examiner variability in detecting OMLs may have led to both under- and over-reporting. In addition, reliance on self-reported data for systemic health and behavioural habits introduces the possibility of social desirability bias and recall inaccuracies. Future research would benefit from longitudinal study designs, more representative sampling strategies, objective clinical assessments and biochemical verification of self-reported data.

## Data Availability

The raw data supporting the conclusions of this article will be made available by the authors, without undue reservation.
